# Mitochondria Autoimmunity and MNRR1 in Breast Carcinogenesis: A Review

**DOI:** 10.33696/cancerimmunol.2.027

**Published:** 2020-12

**Authors:** Félix Fernández Madrid, Lawrence I. Grossman, Siddhesh Aras

**Affiliations:** 1 Department of Medicine, Division of Rheumatology, Wayne State University School of Medicine, Detroit, MI 48201 USA; 2 Center for Molecular Medicine and Genetics, Wayne State University School of Medicine, Detroit, MI 48201 USA; 3 Karmanos Cancer Institute, Wayne State University School of Medicine, Detroit, MI 48201 USA

**Keywords:** MNRR1, Mitochondrial autoimmunity, Autoimmune tissue damage, Chronic inflammation, Breast carcinogenesis

## Abstract

We review here the evidence for participation of mitochondrial autoimmunity in BC inception and progression and propose a new paradigm that may challenge the prevailing thinking in oncogenesis by suggesting that mitochondrial autoimmunity is a major contributor to breast carcinogenesis and probably to the inception and progression of other solid tumors. It has been shown that MNRR1 mediated mitochondrial-nuclear function promotes BC cell growth and migration and the development of metastasis and constitutes a proof of concept supporting the participation of mitochondrial autoimmunity in breast carcinogenesis. The resemblance of the autoantibody profile in BC detected by IFA with that in the rheumatic autoimmune diseases suggested that studies on the autoantibody response to tumor associated antigens and the characterization of the mtDNA- and nDNA-encoded antigens may provide functional data on breast carcinogenesis. We also review the studies supporting the view that a panel of autoreactive nDNA-encoded mitochondrial antigens in addition to MNRR1 may be involved in breast carcinogenesis. These include GAPDH, PKM2, GSTP1, SPATA5, MFF, ncRNA PINK1-AS/DDOST as probably contributing to BC progression and metastases and the evidence suggesting that DDX21 orchestrates a complex signaling network with participation of JUND and ATF3 driving chronic inflammation and breast tumorigenesis. We suggest that the widespread autoreactivity of mtDNA- and nDNA-encoded mitochondrial proteins found in BC sera may be the reflection of autoimmunity triggered by mitochondrial and non-mitochondrial tumor associated antigens involved in multiple tumorigenic pathways. Furthermore, we suggest that mitochondrial proteins may contribute to mitochondrial dysfunction in BC even if mitochondrial respiration is found to be within normal limits. However, although the studies show that mitochondrial autoimmunity is a major factor in breast cancer inception and progression, it is not the only factor since there is a multiplex autoantibody profile targeting centrosome and stem cell antigens as well as anti-idiotypic antibodies, revealing the complex signaling network involved in breast carcinogenesis. In summary, the studies reviewed here open new, unexpected therapeutic avenues for cancer prevention and treatment of patients with cancer derived from an entirely new perspective of breast carcinogenesis.

## Introduction

Recently, Aras et al. reported that MNRR1, a nuclear DNA (nDNA)-encoded mitochondrial antigen, promotes cancer cell migration and the development of metastasis as a proof of concept supporting the participation of mitochondrial autoimmunity in breast carcinogenesis [1]. In this article, in addition to MNRR1, we will review the body of work from our laboratory as well as from the literature showing that other mitochondrial DNA (mtDNA)- and nDNA-encoded mitochondrial antigens are also able to promote breast carcinogenesis and support the view that autoimmune damage to the breast creates a chronic inflammatory environment, fueled by autoantibodies and inflammatory cytokines, that leads to the generation of tumorigenic signals and breast cancer. Since autoimmunity as the engine of carcinogenesis of breast cancer and probably other solid tumors is a novel paradigm not ordinarily linked to carcinogenesis, we have included published and some unpublished work on this new field.

### MNRR1 promotes cancer cell migration and metastasis formation in breast cancer. Proof of concept supporting mitochondrial autoimmunity in breast cancer

A biomarker discovery approach using immunoscreening of a T7 complementary DNA (cDNA) library of breast cancer (BC) proteins with sera from patients with BC containing high titer anti-mitochondrial autoantibodies (AMAs) identified the presence of autoantibodies to Mitochondrial Nuclear Retrograde Regulator 1 (MNRR1; also CHCHD2, AAG10). The presence of MNRR1 was highly associated with the diagnosis of invasive BC [1]. This finding suggested this autoantigen as a promising biomarker of BC that could participate in breast carcinogenesis [1]. In that work, Aras et al. showed that MNRR1, a bi-organellar (mitochondria and nucleus) protein encoded by nDNA, can participate mechanistically in breast carcinogenesis [1]. MNRR1 functions in the nucleus as a transcription factor and in the mitochondria as a regulator of both respiration and apoptosis [2].

MNRR1 is part of a family of proteins marked by the presence of a twin CX_9_C motif (two pairs of cysteines separated by 9 amino acids each). It was predicted to be imported into the mitochondrial intermembrane space via the Mia40/Erv cysteine disulfide relay pathway [3] and Aras et al. showed that it does so [4]. In the mitochondria, MNRR1 interacts with cytochrome *c* oxidase (COX), the terminal complex of the electron transport chain that reduces molecular oxygen to water. Optimal interaction of MNRR1 with COX requires tyrosine 99 (Y99) of MNRR1 to be phosphorylated, a step carried out by Abl2 kinase (Abelson Related Gene, ARG) [5]. Upon interaction, MNRR1 increases COX activity leading to increased ATP generation. MNRR1 has a second role to play in the mitochondria. It also interacts with Bcl-xL and thereby inhibits Bax oligomerization to prevent apoptosis [6].

Aras et al. were the first to show that MNRR1 is also a nuclear localized protein [7]. In the nucleus, MNRR1 binds a core 8-bp DNA element and functions as a transcriptional regulator [4,7] affecting the expression of about 1000 genes. The positively regulated candidate genes belong to critical cellular pathways such as cell growth and migration, mitochondrial function, autophagy, and protein translation. In addition, MNRR1 expression in cells induces an anti-inflammatory phenotype with a reduction in the levels of toxic reactive oxygen species (ROS) [4]. The novel MNRR1 property of being present in two different organelles places it in a regulatory position to control transcriptional activation of target genes in response to cellular redox and energy levels.

Aras et al. and others have shown an increase in MNRR1 transcript levels in BC using both cell lines and patient samples [1,6]. They have also shown using immunohistochemistry that MNRR1 protein levels are higher in BC compared to benign breast disease (BBD) and, importantly, that a knockout of MNRR1 makes cells defective in their migratory capacity [1]. Taken together, these results suggest that MNRR1 mediated mitochondrial-nuclear function promotes BC cell growth and functioning. Thus, the characterization of MNRR1 as a broad mito-nuclear regulator suggests its potential as a novel mechanistic target [1].

Since other mitochondrial autoantigens are potentially downstream of MNRR1, the data presented by Aras et al. strongly support the role of MNRR1 and mitochondrial autoimmunity in breast carcinogenesis. In that work the authors focused on both the direct participation of MNRR1 in breast carcinogenesis and on the demonstration of autoreactivity to this protein in BC sera and its expression in tumor tissue as a proof of concept in support of mitochondrial autoimmunity in BC.

### Anti-mitochondrial antibodies are consistently detected in breast cancer sera

**Figure 1:**
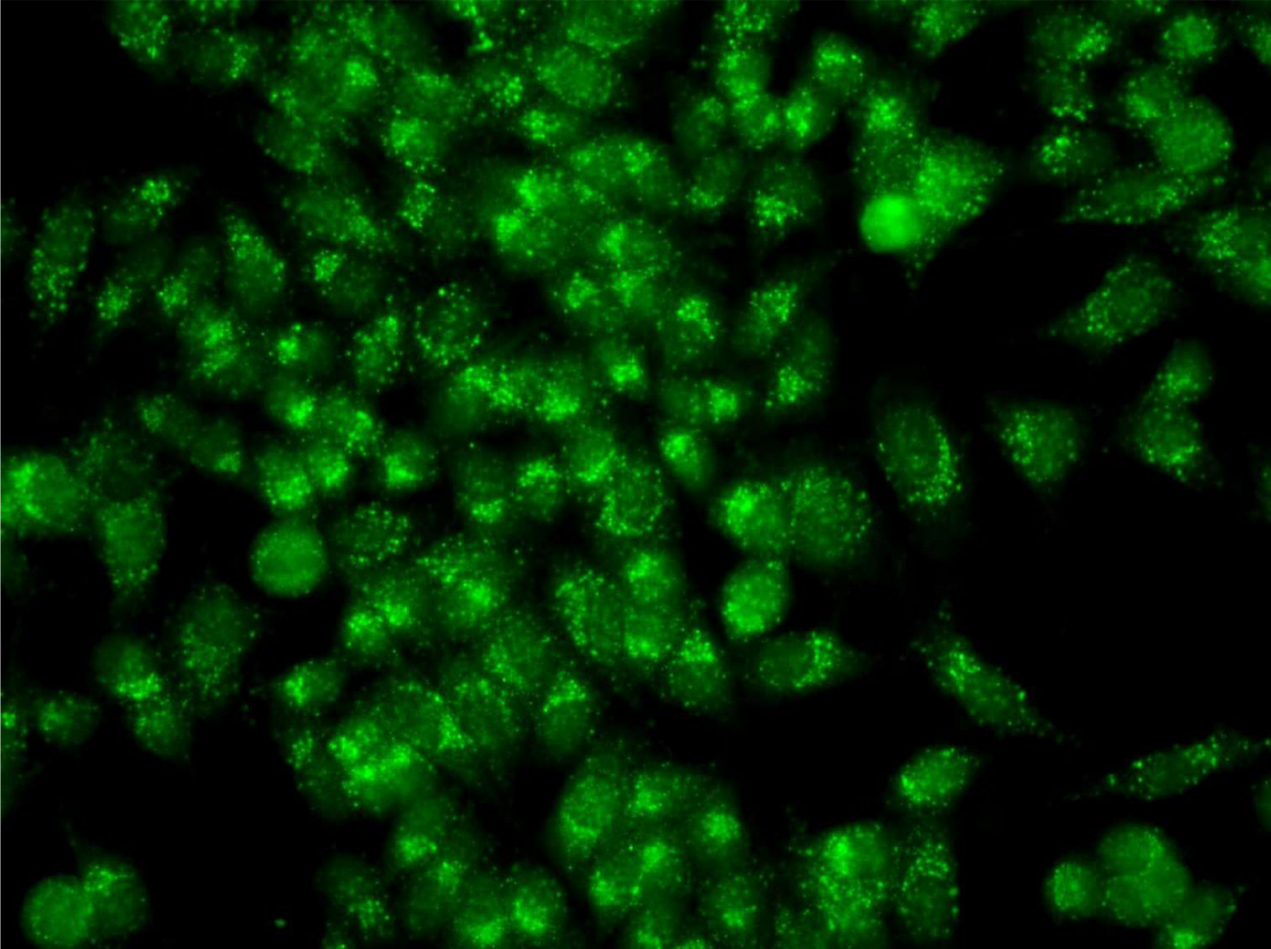
Mixed IFA pattern with antibodies to mitochondria and centrosomes in the serum of a patient with ductal carcinoma *in situ.*

**Figure 2:**
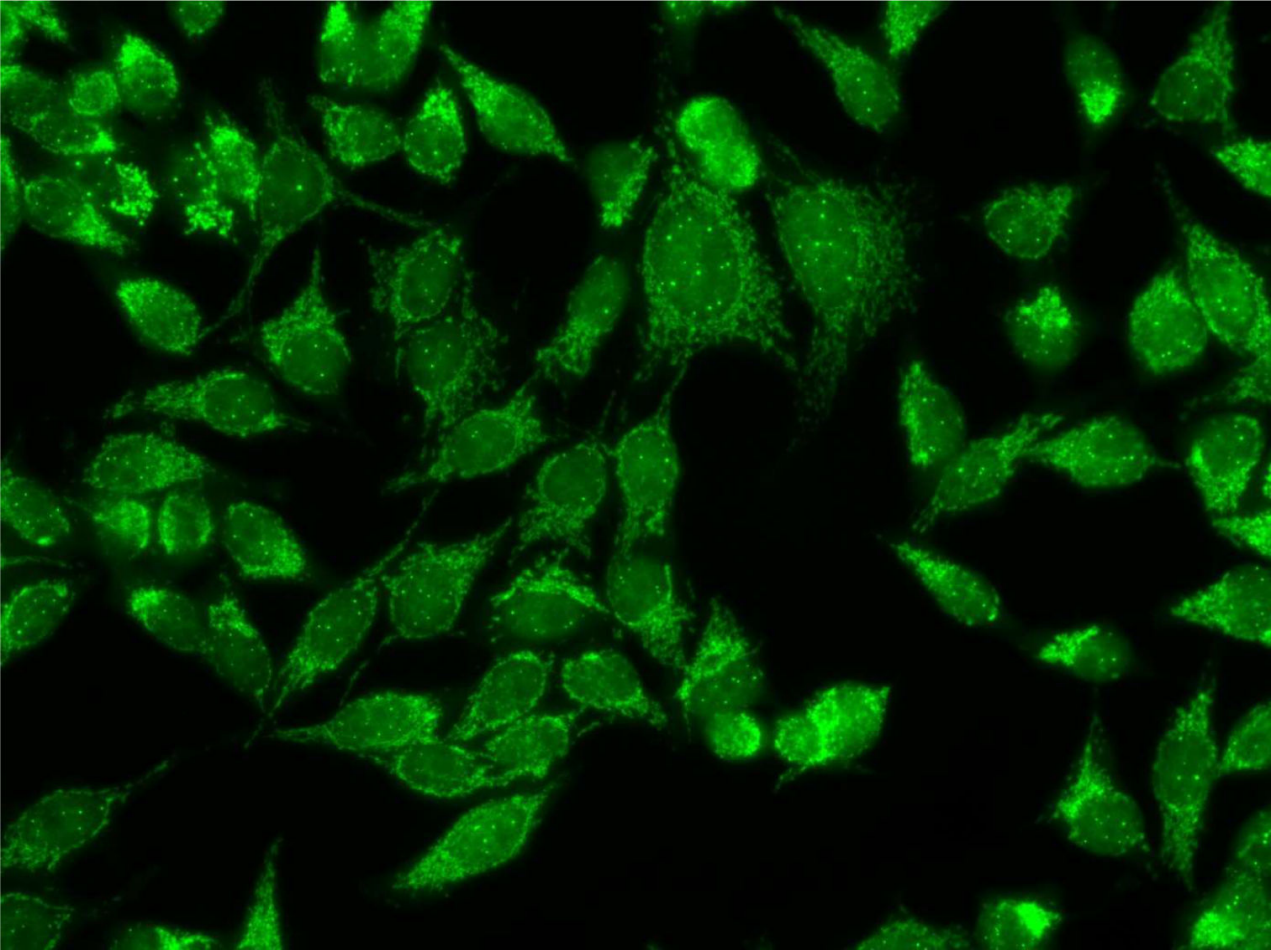
AMAs in the serum from a patient with Infiltrating breast carcinoma.

Anti-mitochondrial antibodies (AMAs) are found consistently in the sera from patients with primary biliary cholangitis (PBC) and pemphigus vulgaris (PV) [11,12] and occasionally in some of the rheumatic autoimmune diseases (RADs) but they had not been found in the serum from patients with any other disease [10] prior to the report of AMAs in the sera from patients with infiltrating ductal breast carcinoma (IDC) and in ductal carcinoma *in situ* (DCIS) [8,9]. Immunofluorescence (IFA) is a time-honored method to detect AMAs in the clinical setting and the mitochondrial pattern detected by IFA (Figures 1 and 2) has been the gold standard to document the presence of AMAs in primary biliary cholangitis and in pemphigus vulgaris in the clinical laboratory. That the mitochondrial pattern detected by IFA is related to AMAs has been validated by multiple studies [Reviewed in 10–13].

AMAs are found in serum from patients with PBC, an autoimmune liver disease [11], and in the sera of patients with PV, an autoimmune skin disease caused by a loss of epidermal cohesion and manifested by progressive blistering and non-healing erosions [12]. Autoimmunity has been implicated in the pathogenesis of both these diseases. In addition, there is some evidence to indicate that autoantibodies generated via somatic mutations play a role in producing loss of keratinocyte cell adhesion in PV [13].

**Figure 3:**
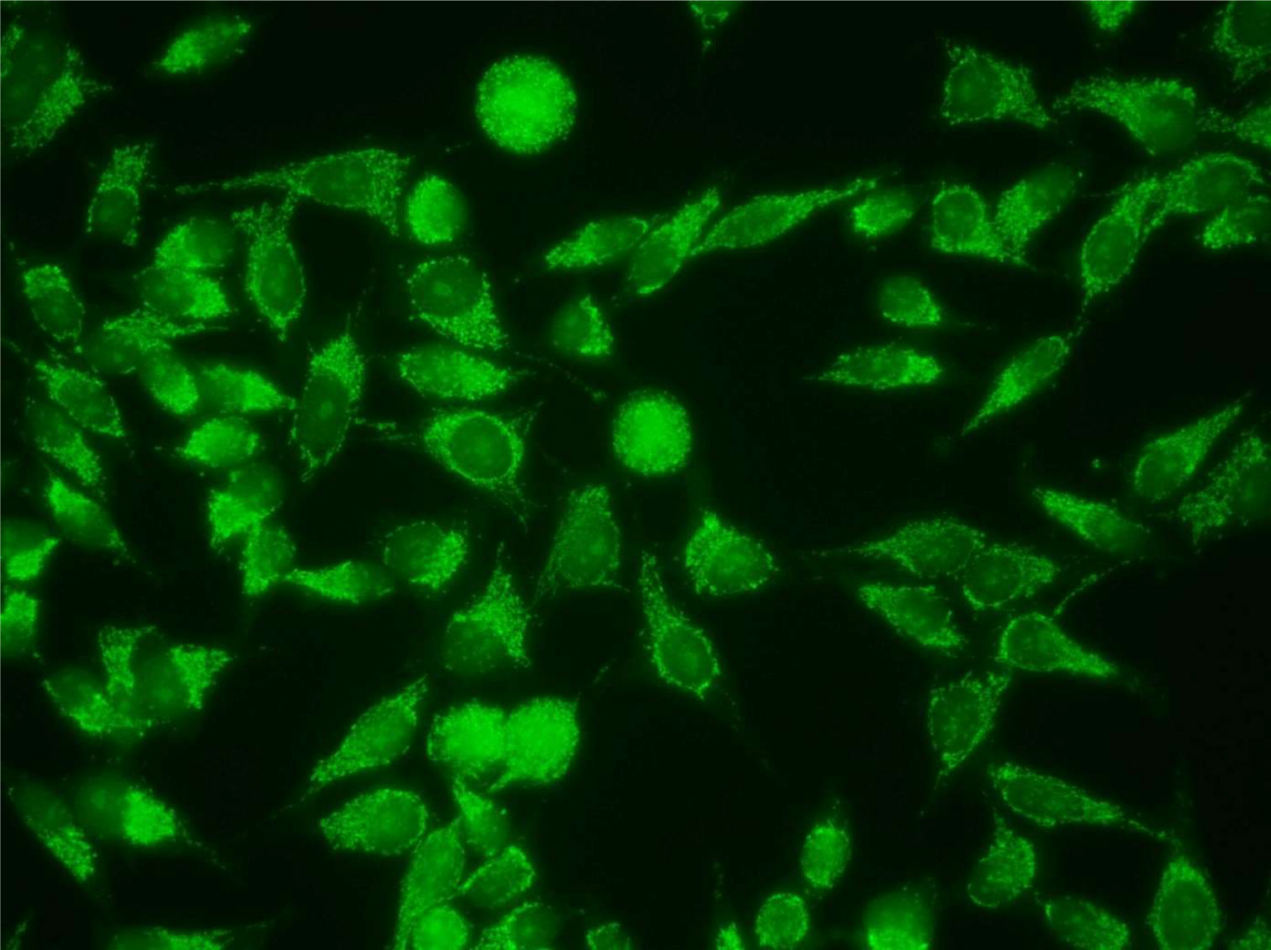
Mixed pattern with AMAs and nucleolar staining in serum from a healthy woman with BIRADS4 mammography assessment and BBD by breast biopsy.

A comprehensive study of autoantibodies performed using IFA on a collection of sera from women with BC or benign breast disease (BBD) undergoing annual screening mammography detected autoantibodies in virtually all patients with BC, predominantly of the IgG1 and IgG3 isotypes [9]. AMAs were a prominent finding in the autoantibody profile detected in BC sera, which showed distinctive features including antibodies also targeting centrosomes, centromeres, nucleoli, and the cytoskeleton that are thought to be the expression of tumor immunogenicity. The majority of BC sera showing AMAs did not react with the M2 component of pyruvate dehydrogenase, characteristic of PBC; thus, the data suggested the involvement of a different set of mitochondrial antigens in BC. This study concluded that autoantibodies developed in BC are not an epiphenomenon, but likely reflect an antigen-driven autoimmune response triggered by epitopes developing in the mammary gland during breast carcinogenesis [9]. High-titer autoantibodies targeting predominantly mitochondria but also centrosomes and centromeres were also detected in a small group of healthy women with suspicious mammography assessment and BBD but not malignancy by breast biopsy, suggesting that the process inducing autoantibody formation starts in the pre-malignant phase (Figure 3). The mitochondrial pattern by IFA reflecting the presence of AMAs is rare in the general population [10] and when it is found may suggest primary biliary cholangitis or pemphigus vulgaris, the only two diseases that were known to present AMAs before the reports of AMAs in BC [8,9,17], suggesting that healthy persons in the general population do not have these antibodies unless they have asymptomatic PBC or may be predisposed to BC.

The frequent finding of typical AMA patterns in healthy women found to have BBD at breast biopsy suggested that a panel of autoantigens targeted by AMAs might allow detection of BC risk in asymptomatic women [9]. This finding is particularly notable in view of the rarity in the general population of the mitochondrial pattern observed by IFA [10].

In this vein, a collaborative study of Michael P Long at Wayne State University, Azadeh Stark at Henry Ford Health System (HFHS), and Sasi Mudumba at Genalyte, San Diego CA (database updated as of June 2018) discovered that, during a follow-up of median 9.1 years of a small cohort of 233 healthy women undergoing annual screening mammography at HFHS who had BIRADS4 assessment and BBD at breast biopsy, a total of 17 women (7.3%) were diagnosed with malignant carcinomas: 8 (3.4%) were diagnosed with BC and 9 (3.9%) with other malignancies including lung, pancreatic, ovarian, and small intestinal cancers or lymphoma. The analysis of autoantibody profile of the sera obtained at the time of the initial breast biopsy that included four different panels of mitochondrial and non-mitochondrial antigens using a novel multiplexed platform, the Maverick Detection System, based on silicon photonics, indicated that these women who were healthy at the time of the breast biopsy, and were not known to be at risk for BC, had an abnormal autoantibody profile and that the diagnosis of malignancy could have been made or suspected several years before the actual clinical diagnosis. For comparison, an American woman with the life expectancy of 78 years has about a 38.70% risk of developing an invasive malignant condition during her lifetime or about 0.496% per year of life. In other words, annually approximately 500 of 100,000 American women are at a risk of being diagnosed with an invasive malignancy (American Cancer Society) whereas the estimated annual incidence or risk of experiencing a malignant tumor in the cohort of women who contributed to this study was 0.802%, or 802 per 100,000 women, which is 1.61 fold (95% CI 1.44–1.80, P<0.001) higher than the estimated annual risk for an American woman. This result suggests that healthy women undergoing annual screening mammography with BIRADS4 assessment and BBD and no cancer at breast biopsy might be at an increased risk for malignancies and may merit a closer follow up [Unpublished]. This study also suggests that a panel of mitochondrial and non-mitochondrial antibodies has screening potential for breast and possibly other malignancies. These suggestions must be confirmed and deserve further investigation.

The resemblance of the autoantibody profile in BC with that in the RADs such as systemic lupus erythematosus (SLE) and rheumatoid arthritis (RA) by IFA [9] suggested that studies on the autoantibody response to tumor associated antigens (TAAs) and the characterization of the mtDNA- and nDNA-encoded antigens forming the mitochondrial autoreactome in BC may provide functional data on breast carcinogenesis.

### Approach to identify a mitochondrial autoreactome

The autoimmune nature of the molecular changes observed in PBC and in PV [11,12], the evidence that autoimmunity is responsible for the direct participation of autoreactive MNRR1 in breast carcinogenesis [1], and the report of consistent mitochondrial autoreactivity detected by IFA in BC sera [9], suggested that a comprehensive survey of mitochondrial autoreactivity in BC could reveal a wealth of diagnostic and prognostic biomarkers. Following the seminal work of E.M. Tan on autoimmunity in the RADs [14], it was expected that the mitochondrial and non-mitochondrial autoreactome in BC could provide functional information on breast carcinogenesis.

The basic approach used for biomarker discovery – to isolate and characterize the putative TAAs, including the construction of cDNA expression libraries, immunoscreening of cDNA libraries of potential BC autoantigens, the assembly of micro-collections of the cloned phages on derivatized glass slides, hybridization of the cloned phages with sera from cases and non-cancer sera, development of the autoantigen microarray, identification of unique clones using PCR, cDNA sequence determination, and homology searches of informative phages in databases – have all been reported [15–17] and were adapted to identify the mitochondrial autoreactome in BC.

Sera containing autoantibodies of unknown specificity had been used successfully in previous works for immune screening the cDNA library of potential BC autoantigens. However, in experiments aimed to identify BC biomarkers, the use of biopanning BC sera containing an abundant unknown 56 kDa antigen led to the identification of Annexin XIA as a prominent BC autoantigen [16]. Based on this successful experience to promote the identification of certain antigens, the approach used to facilitate the identification of mitochondrial autoantigens was to use BC sera containing high titer AMAs (≥ 1:320–640 dilution) detected by IFA for biopanning the cDNA libraries [17].

The reports suggesting that epithelial cancer cells participate in immunoglobulin synthesis [18–22] prompted the use of two different cDNA expression libraries to characterize the mitochondrial autoreactome, a random primer multi-human BC cell line library (RP library), and a cDNA library (B library) of commercial origin (Novagen). These two cDNA libraries were used to differentiate BC antigens generated within epithelial cancer cells without the influence of the microenvironment and the immune cells (RP library) from those generated in the whole tumor tissue (B library) [17].

Since commercially obtained libraries are usually derived from a single malignant tumor, and given the heterogeneity of BC [23], a multi-human BC cell line cDNA library was constructed by directional cloning of randomly primed cDNA from 7 human breast carcinoma cell lines: SUM 44, SUM 102, SUM 149, SUM 159, MCF7, SKBR, T47D and one BC pre-malignant cell- the DCIS.com cell line into a T7 phage display vector using T7 Select 10–3b vector and the Orient Express cDNA library construction system (Novagen, Billerica, MA, US) as reported [17]. The insert size of individual clones of the complete library was analyzed by PCR. Biopanning with BC sera containing high titer AMAs was followed by microarray assembly of the identified clones, hybridization of the potential BC autoantigens with sera from cases of BC, and non-cancer controls with exclusion of the sera used for biopanning the cDNA libraries, and development of the autoantigen microarray as reported [15–17]. Sequence determination of phage inserts from informative phages (Genewiz, South Plainfield, NJ) was followed by homology searches in databases using BLAST and BLAT for antigen identification [24,25].

### mtDNA-encoded enzyme components of the electron transport chain are targeted by anti-mitochondrial antibodies

The evidence for the direct participation of MNRR1 in breast carcinogenesis and the demonstration of autoreactivity to this protein in BC sera constitute proof of concept that MNRR1 is involved in BC progression and metastasis, supporting the involvement of autoimmunity in breast carcinogenesis [1]. Nevertheless, nDNA-encoded MNRR1 is not the only mitochondrial protein involved in breast carcinogenesis. The components of the mitochondrial autoreactome are encoded by both mtDNA and nDNA. Maroun et al. recently reported that several mtDNA-encoded enzyme components of the electron transport chain (ETC) are targeted by autoantibodies in BC sera and proposed that the broad autoreactivity to mitochondrial antigens is the expression of autoimmunity in BC [17]. Initial work to characterize the mitochondrial autoreactome in BC validated the serum mitochondrial autoreactivity previously detected by IFA [8,9] by identifying the mitochondrial gene products recognized as foreign by the immune system. Among the clones selected by immune-screening, a group of phage inserts was found expressing in-frame protein components of complexes I, IV, and V of the ETC encoded by mtDNA. Cloned sequences included multiple phage inserts identical to NADH-ubiquinone oxidoreductase subunit ND5, and one clone each identical to ND4, ND6, ATP synthase subunits MT-ATP6 and MT-ATP8, cytochrome *c* oxidase subunit MT-CO1 [26,27], and to the RNA gene MT-RNR2 [17].

A novel finding revealed by the mitochondrial autoreactome in BC was that AMAs can target and significantly recognize sequences in mitochondrial RNA genes [17]. This finding was unexpected because autoantigen microarrays have been widely used to identify expression sequence tags (ESTs) derived from protein coding genes. However, cDNA libraries have the capability of detecting autoreactive RNA clones since Poly(A) RNA is used for their construction. There is evidence that antibodies provide an effective tool for detecting RNA conformation [28] and there are many examples of RNA sequences recognized by anti-RNA antibodies in the RADs and in viral infections [29–32]. Recently, there has been increasing evidence supporting that 16S mitochondrial ribosomal RNA is involved in coding for small peptides such as Humanin [33]. In addition to servicing the cell by producing ATP energy and regulating apoptosis in response to complex signals, mitochondria fulfill other vital roles such as communicating back to the nucleus in determining cellular policies [33]. Some of these retrograde signals are encoded by mtRNA, and consist of a family of small peptides, the products of mitochondrial metabolism [33–38]. In the work of Maroun et al. three clones of MT-RNR2 were identified, two of them chimeric and one non chimeric [17]. This latter clone encompassed a sequence encoding the small mitochondrial peptide SHLP2 [37]. Although the significance of the chimeric clones is uncertain, the three clones with ESTs possibly encompassing SHLP2 sequences were significantly recognized by BC sera on the microarray [17].

Mitochondrial transcriptome analyses have shown that multiple small mRNAs are transcribed from mtDNA. Cobb et al. identified six small open reading frames (ORFs) in the MT-16S rRNA region labeled small humanin-like peptides (SHLPs) 1−6, that are 24–38 amino acids in length [34]. Of these, SHLP2 is a 26 amino acid peptide that has been shown to induce mitochondrial biogenesis in pancreatic cancer cell lines [34]. Mitochondrial-derived peptides have been proposed to provide metabolic properties, and neuroprotective, cytoprotective, anti-oxidant, and anti-inflammatory effects [33–38]. It has been demonstrated that SHLP2 mediates chaperone-like effects [38]. Assuming that the directionality in the reported non chimeric clone [17] is sense, the autoreactive sequence would correspond to SHLP2, which resides entirely within the region covered by the autoreactive sequence. All the other small mitochondrial peptides including Humanin were found to reside outside of the mtDNA region covered by the three clones. If the finding that SHLP2 is targeted by AMAs in BC is confirmed, it is possible that this small mitochondrial peptide may have a role in breast carcinogenesis through its regulatory activity on apoptosis, insulin sensitivity, and inflammation.

It is clear that the AMAs present in BC sera targeting key components of the ETC and mitochondrial 16S RNA are features of the autoantibody profile not found in other diseases reported to be associated with AMAs such as PBC [11] or PV [12]. It is notable that ND4, ND5, and ND6 targeted by AMAs are highly associated with the diagnosis of BC since they are core subunits of the mitochondrial membrane respiratory chain NADH dehydrogenase (complex I) that constitute the minimal assembly required for catalysis [17]. Complex I functions in the transfer of electrons from NADH to the rest of the respiratory chain; thus, autoreactivity involving the components of complex I suggests that this process, although not necessarily the proximal cause of cancer, may be impaired in some cases of BC.

Sequence variants in MT-ATP6, the D-loop, MT-ND3, ND5, and ND6 have been reported to make important contributions to glucose and insulin metabolism, adipocyte regulation, diabetes, and cardiovascular disease [39]. Primary breast tumors harbor somatic mtDNA variants with possible functional consequences. RNA sequencing studies of primary BC demonstrated that somatic variants detected at the mtRNA level are representative for somatic variants in the mtDNA and that the number of somatic variants within the mitochondrial transcriptome is positively associated with age at diagnosis of BC although the exact impact on metabolism and clinical relevance are not known [40].

The MT-ATP8 gene encodes a subunit belonging to the proton channel of ATP synthase. ATP synthase generates ATP against a proton gradient in response to electron transport in the mitochondria. The relevance of autoantibodies to a gene product of MT-ATP8 in BC is suggested by the study of McGeehan et al. who reported mtDNA variations including MT-ATP8 in blood samples derived from a small group of women who were diagnosed with early-onset BC and later went on to develop breast to brain metastasis [41]. Further research into the significance of the autoreactivity of mitochondrial ribosomal RNA associated with the diagnosis of BC is warranted, in particular in relation to the role of small mitochondrial peptides encoded by 16S mitochondrial ribosomal RNA. The finding of autoreactive SHLP2 in BC sera may be evidence that the process involving the 16S mitochondrial ribosomal RNA in coding for small mitochondrial peptides is disturbed in BC and can have functional consequences to be determined.

### Mitochondrial autoimmunity may be the link between mitochondrial somatic mutations and BC

Maroun et al. [17] speculated that the immunogenicity of the enzyme components of complex I is related directly or indirectly to their relative enrichment in somatic mutations [42,43]. In support of that proposal, a direct involvement was suggested by several studies reporting an association between mitochondrial somatic mutations and autoimmunity [44,45]. It has been demonstrated that peptides of mutated or aberrantly expressed mitochondrial proteins can be recognized by the immune system. Chen et al. tested the hypothesis that altered self-proteins translated from mtDNA somatic mutations play a role in the development of autoimmunity, suggesting that mtDNA mutations may trigger immune responses [44]. This group provided proof of principle that the immune system can recognize peptides generated as a result of spontaneous somatic mutations. Gu et al. reported that damaged mtDNA is associated with increased expression of class I MHC, providing evidence that mutated peptides derived from somatic mutations in mtDNA can be recognized by the immune system [45]. However, reports of conflicting data [reviewed in 46,47] suggest that the relationship between mitochondrial somatic mutations and carcinogenesis and/or cancer progression, although compelling, is not straightforward. Indeed, although mitochondrial somatic mutations are prevalent in BC and other cancers, the complexity of carcinogenesis has been recognized [48]. Although mutated proteins can be recognized by the immune system as non-self and generate autoantibody responses [44,45], structural changes in mitochondrial antigens as a consequence of mutations may not cause most of the immunogenicity of the gene products targeted by AMAs in BC since the autoantibody response observed in cancer sera as well as in the sera of patients with RADs targets both mutated and wild type proteins [14,49,50].

Nevertheless, the demonstration of the direct participation of MNRR1 in breast carcinogenesis, and the autoreactivity to this protein in BC sera and its expression in tumor tissue, constitute a proof of concept in support of mitochondrial autoimmunity in BC [1]. The report of AMAs in BC sera resembling features typically found in the autoimmune RADs [9] and the report showing that autoreactivity of the enzymes of the ETC is a feature of BC [17], are highly supportive of an important role of mitochondrial autoimmunity in BC. In addition, the reports of a strong association between somatic mitochondrial mutations and BC progression [42,43,46,47], and the studies of the immunogenicity of mitochondrial gene products [44,45], suggest a link between mitochondrial autoimmunity and somatic mutations, although this link may not be a direct one.

It is likely that somatic mutations are causally related to mitochondrial autoreactivity without responding to structural changes in the protein. A clue comes from the reports of an association between mitochondrial somatic mutations and the unfolded protein response (UPR) [51,52]. Alterations in ER homeostasis can cause accumulation of misfolded/unfolded proteins in the ER as well as in the mitochondrial matrix. To maintain ER/mitochondrial matrix homeostasis, eukaryotic cells have evolved highly specific signaling pathways to ensure that their protein folding capacity is not overwhelmed. The UPR is an essential adaptive intracellular mechanism adopted by the cell to survive the ER stress. Proteins that are misfolded in the ER are retained until they reach their native conformation or are retro-translocated back into the cytosol for degradation by the 26S proteasome. The UPR has been implicated in a variety of metabolic, neurodegenerative, and inflammatory disorders, and is known to be activated in cancers including BC [51,56]. It is possible that the link between somatic mutations and BC progression resides in ER and mitochondrial matrix stress caused by somatic mutations producing the accumulation of unfolded or misfolded proteins. Multiple studies have shown somatic mutations activating ER-stress and triggering the UPR [51,52]. Since mitochondria have their own protein synthesizing machinery [57], accumulation of misfolded proteins in the mitochondrial matrix could occur as a consequence of stress caused by mitochondrial somatic mutations; in addition, ROS are increased in solid tumors [58–60], and therefore it is possible that exposure to excessive oxidative insult is an additional factor in producing ER and mitochondrial stress. Todd et al. have discussed the growing evidence that dysregulation of the UPR can participate in the development of autoimmunity [61]. Although it is also possible that direct ROS damage to mtDNA and mitochondrial proteins, which undoubtedly can occur [58,59], contributes to the immunogenicity of mitochondrial proteins in cancer cells, there is no evidence in support of this possibility in BC. The hypothesis of a causal relationship between mitochondrial somatic mutations and mitochondrial stress induced autoimmunity [17] needs to be examined further. It was suggested that an in-depth study of mitochondrial autoimmunity in patients with BC as well as in pre-malignant breast tissue may result in the identification of invaluable diagnostic and prognostic biomarkers for early aggressive BC [17].

Relevant to the proposal that autoimmunity may be the engine that promotes breast carcinogenesis [9,17], there is an established link between chronic inflammation and the generation of solid tumors [62,63]. Chronic inflammation as a result of autoimmune breast tissue damage may provide a rationale for the reported paradoxical observation of B-cell hyperactivity and BC progression [64,65].The proposed model of cancer progression based on mitochondrial autoimmunity implies a vicious circle of mitochondrial and ER stress, immune recognition of accumulated unfolded or misfolded proteins by autoreactive immune cells, autoimmune damage of the target organ, and chronic inflammation with generation of protumoral signals. Misfolded proteins could be recognized as non-self by auto-reactive immune cells in the context of MHC class II molecule in BCs, but other possibilities linking autoimmunity with UPR [61] could also explain, at least in part, the generation of mitochondrial autoimmunity. It is likely that the paradigm shift in cancer progression that recognizes the tumorigenic effect of autoimmunity-mediated breast tissue damage resulting in chronic inflammation may result in novel therapeutics for BC.

### Autoreactive nDNA-encoded mitochondrial proteins contribute to breast cancer progression and metastases

The finding that MNRR1 is targeted by AMAs in multiple BC sera offered the opportunity to investigate the participation of this nDNA-encoded mitochondrial antigen in breast carcinogenesis [1]. In addition to autoreactive MNRR1 in BC, other nDNA-encoded mitochondrial autoantigens have been reported in the past in association with the diagnosis of BC. The use of immune-screening cDNA libraries and autoantigen microarray analyses led to the identification of PRAX-1 as a BC autoantigen associated with the diagnosis of invasive breast carcinoma [15,16]. The peripheral-type benzodiazepine receptor (PBR) is a widely distributed transmembrane protein located in the outer mitochondrial membrane. Multiple functions have been attributed directly or indirectly to the PBR, including the regulation of cholesterol transport and the synthesis of steroid hormones, porphyrin transport and heme synthesis, apoptosis, cell proliferation, anion transport, regulation of mitochondrial functions, and immunomodulation [66]. Based on these varied functions, it was proposed to re-name PBR as translocator protein (18 kDa), regardless of the subcellular localization of the protein, to represent more accurately its subcellular roles and putative tissue-specific functions [67]. The PBR has been found associated with cell proliferation in aggressive BC cell lines and glioma cells [68].

The characteristics of the nDNA-encoded mitochondrial antigens, and the autoreactive signal transduction molecules interacting with the nDNA-encoded mitochondrial proteins putatively involved in breast carcinogenesis revealed by immune-screening cDNA libraries followed by autoantigen microarray analysis, are included in Table 1 [Deya Obaidat et al., unpublished].

#### The mitochondrial autoreactome in breast cancer suggests that DDX21 orchestrates a complex signaling network with participation of JUND and ATF3 driving chronic inflammation and breast tumorigenesis:

Fourteen clones of the DEAD box protein DDX21 were identified in the RP cDNA library that were significantly associated with BC (Table 1). DDX21 is a nucleolar protein involved in RNA processing. In addition to DDX21, the proto-oncogene JUND and AFT3 were recognized as autoantigens by BC sera by immune-screening and microarray analyses (*p* for BC = 0.0001 and 0.02, respectively) [Obaidat et al., unpublished]. Zhang et al. reported that DDX21 is highly expressed in BC tissue and in established cell lines, its protein expression levels correlate with cell proliferation rate, and it is induced by *EGF* signaling [69].

Activated PARP-1 ADP-ribosylates DDX21 and promotes rDNA transcription [70]. Elevated levels of PARP-1 and DDX21 are also associated with cancers. DDX21 expression in BC cells can promote tumorigenesis via effects on AP-1. Mechanistically, DDX21 is required for the phosphorylation of c-Jun, and DDX21 deficiency markedly reduces the transcriptional activity of AP-1 [69].

It is revealing that AP-1 itself is also targeted by autoantibodies in BC since the JUND Proto-Oncogene AP-1 Transcription Factor Subunit was cloned from the B library while ATF3 was cloned from the RP cDNA library and both were significantly recognized by BC sera. The *ATF3* gene encodes a member of the mammalian activation transcription factor/cAMP responsive element-binding (CREB). This stress-responsive, cyclic AMP-dependent transcription factor is also involved in tumorigenesis. Relevant to the possible effect of autoreactive ATF3 in BC, this transcription factor interacts with ERK/MAPK signaling and with pathways involved in glucose/energy metabolism that are involved in carcinogenesis [71]. The involvement of ATF3 in breast carcinogenesis has attracted a great deal of attention [72,73]. *ATF3*, an adaptive-response gene, enhances TGFβ signaling and cancer-initiating cell features in BC cells. The study of Zhang et al. also reported that elevated DDX21 regulates c-JUN activity and rRNA processing in human BC [69]. JUND is at the crossroads of several key tumorigenic pathways such as the IL-17, IL-1, MAPK3, IL-2, and the Ras family of GTPases [74], suggesting the prominent involvement of this complex signaling network orchestrated by DDX21 in chronic inflammation in BC. The data showing autoreactivity of DDX21, JUND, and ATF3 are in agreement with the suggestion that ATF3 has pro-tumorigenic activity in BC [72,73].

#### Key mitochondrial enzymes involved in energy metabolism are targeted by autoantibodies in breast cancer:

Almost a century ago Otto Warburg observed that cancer cells, unlike many normal cells, rely on glycolysis rather than on mitochondrial respiration, even in the presence of oxygen. This phenomenon, confirmed by many investigators ever since, became known as the Warburg effect [75]. Warburg attributed the aerobic glycolysis phenotype to irreversible mitochondrial dysfunction. He proposed that dysfunctional mitochondria are required to start all the biochemical events that eventually lead to cancer formation [75]. However, Warburg’s proposal is controversial since subsequent studies showed functional mitochondria in cancer cells [76,77]. The opposing view was based on the finding that cancer cells are often able to oxidize glucose and fatty acids to carbon dioxide at levels comparable to those of normal cells, i.e., that cancer cells have reduced mitochondrial activity as a consequence of heightened glycolytic flux, which they use to provide growth precursors such as pyrimidines; glycolysis is known to inhibit mitochondrial respiration. This controversy has recently been reviewed by Senyilmaz and Teleman [78]. In relation to this issue, autoreactive GAPDH was identified in the RP cDNA library by immune-screening and microarray analyses (Table 1). GAPDH enzyme catalyzes the reversible oxidative phosphorylation of glyceraldehyde-3-phosphate in the presence of inorganic phosphate and nicotinamide adenine dinucleotide (NAD), thereby playing a critical role in glycolysis. GAPDH has many non-metabolic functions that could promote tumorigenesis. The protein also exhibits nitrosylase activity as evidenced by cysteine S-nitrosylation of nuclear target proteins such as SIRT1, HDAC2, and PRKDC, thereby regulating nuclear functions including transcription, RNA transport, DNA replication, and apoptosis [79]. Importantly, this enzyme modulates the organization and assembly of the cytoskeleton. GAPDH is a component of the GAIT (gamma interferon-activated inhibitor of translation) complex, which mediates interferon-gamma-induced inhibition of translation in pathophysiology of inflammation [80]. In response to interferon gamma treatment, GADPH assembles into a complex (GAIT) to interact with GAIT elements in the 3’-UTR of diverse inflammatory genes and suppresses their translation. This protein also harbors a peptide that has antimicrobial activity against bacteria found in the human fecal microbiome.

GAPDH expression is associated with BC cell proliferation and with the aggressiveness of tumors [81]. Guo et al. proposed that GAPDH plays an important role in carcinogenesis through the regulation of the cell cycle based on the demonstrated altered GAPDH gene expression during growth in different types of tumors such as breast, lung, renal, gastric, glioma, liver, colorectal, prostatic, pancreatic, and bladder cancers and in melanoma [82]. The suggestion that GAPDH plays an important role in carcinogenesis through the regulation of the cell cycle is supported by the report that this enzyme, as well as a group of important centrosome antigens associated with centrosome assembly and/or microtubule function such as stathmin 1, SUMO/Centrin peptidase, peri-centriolar material-1, HS actin gamma1, and ubiquitin-conjugating enzyme E2, are targeted by autoantibodies in BC sera, all of them associated with the diagnosis of invasive BC [83]. The finding that some of these antibodies are present in a group of healthy women with suspicious mammography findings (BIRADS4 assessment) [9] suggests that breakdown of tolerance to centrosome proteins occurs early in breast carcinogenesis and that autoantibodies to centrosome and mitochondrial antigens might be biomarkers of early BC [83]. In agreement with this suggestion, the translocation of GAPDH from the cytoplasm, probably from the mitochondria in quiescent cells to the nuclear or perinuclear regions of proliferating cells, suggested a functional role of GAPDH in cell growth [84], and the mRNA and protein levels of GAPDH were shown to be dependent on the cell cycle [85]. The enhanced expression of GAPDH can be accompanied by an increase in the levels of enolase and glucose transporter and an accelerated rate of glucose transport, which has long been known to accompany cellular transformation [86]. It has been suggested that up-regulation of GAPDH could contribute to an augmented rate of glycolysis in tumor cells and/or maintenance of a transformed phenotype [87]. Growth factors such as insulin and epidermal growth factor could increase the expression of GAPDH in some cell lines by promoting tumor growth.

In aggregate, these reports support the view that autoimmunity plays a role in deregulating GAPDH [81] that might be involved in invasiveness and metastasis of BC and possibly of certain other solid tumors through its participation in the glycolysis pathway and/or cell cycle events. Autoimmunity depicts a faithful portrait of the major players involved in breast carcinogenesis that may be valuable diagnostic and prognostic biomarkers of early BC. In addition, the marked increase in GAPDH expression associated with the rapid growth of cancer cells suggests that suppression of this key reaction in glycolysis could have therapeutic value as a major strategy to control breast and other human cancers.

#### Pyruvate kinase (PKM), one of the main enzymes involved in glycolysis, is targeted by autoantibodies in BC:

There is evidence that PKM2, a glycolytic enzyme that catalyzes the transfer of a phosphoryl group from phosphoenolpyruvate to ADP, generating ATP, is a BC autoantigen (Table 1). PKM induces POU5F1-mediated transactivation, which plays an important role in stem cell pluripotency. Perhaps related to this activity of PKM, it has been reported that cancer stem cells play an important role in metastasis. Zhao et al. reported a specific role of PKM2 in the stemness of BC cells [88]. PKM plays a role in caspase independent cell death in tumor cells. The ratio between the highly active tetrameric form of PKM and the dimeric form determines whether glucose carbons are channeled to biosynthetic processes or used for ATP production. The transition between PKM1 and PKM2 contributes to the control of glycolysis and is important for cell proliferation and survival. Reprogramming of cell metabolism regulated by a complex signaling network in which PKM2 plays a critical role, is essential for tumorigenesis [89]. While dimeric PKM2 diverts glucose metabolism towards anabolism through aerobic glycolysis, tetrameric PKM2 promotes the flux of glucose-derived carbons.

PKM2 is upregulated in BC tissue and high PKM2 levels were reported to be associated with poor prognosis of BC patients [88].

Equilibrium of the PKM2 dimers and tetramers is critical for tumorigenesis and is controlled by multiple factors. As discussed above, autoantibodies in BC sera also target GAPDH, a key enzyme of glycolysis. It has been suggested that PKM2 functions independently of its pyruvate kinase activity, which is crucial for cancer cell proliferation [90]. Hsu et al. discussed growing evidence indicating that dimeric PKM2 is released from tumor cells into the circulation of cancer patients and revealed a novel function of extracellular PKM2 in promoting cancer cell proliferation through EGFR activation. In addition to the role of PKM in carbohydrate metabolism, this protein may mediate cellular metabolic effects induced by thyroid hormones and may have a role in bacterial pathogenesis.

In summary, the finding that this multifunctional protein and other important synergistic proteins involved in the metabolism of carbohydrates and in BC progression and metastasis such as GAPDH are targeted by autoantibodies in BC (Table 1) supports the dominant participation of autoimmunity to multiple TAAs in breast carcinogenesis.

#### Autoantibodies to GSTP1 in breast cancer sera recognize a susceptibility factor that may be involved in breast carcinogenesis:

Three clones of glutathione S-transferase Pi 1 identified in the RP cDNA library (Table 1) were significantly recognized by BC sera. Glutathione S-transferases are a family of enzymes that play an important role in detoxification by catalyzing the conjugation of hydrophobic and electrophilic compounds with reduced glutathione. The GSTP1 gene encodes active, functionally different proteins that play a role in susceptibility to cancer and other diseases via regulation of xenobiotic metabolism. GSTP1 is commonly inactivated by somatic CpG island hypermethylation in cancers of the prostate, liver, and breast. Lin and Nelson reported that hypermethylation of CpG dinucleotides at the 5’ transcriptional regulatory region was sufficient to inhibit GSTP1 transcription in MCF-7 BC cells and that repression of GSTP1 transcription was mediated in part by the methyl-CpG binding domain protein MBD2 [91].

Glutathione S-transferase Pi is a susceptibility factor in esophageal and prostate cancers. GSTP1 gene polymorphism increases age-related susceptibility to hepatocellular carcinoma [92]. Polymorphism of the *GSTP1* gene seems associated with elevated BC risk in a race-specific manner [93]. c-Jun N-terminal kinase (JNK)-mediated cell signaling pathways are regulated endogenously in part by protein-protein interactions with GSTP1. Wang et al. provided evidence for direct interaction between the C-terminus of c-Jun N-terminal kinase JNK and GSTP1–1 and a rationale for considering GSTP1–1 as a critical ligand-binding protein with a role in regulating kinase pathways [94]. JNK is a member of the mitogen activated stress kinase family (MAPK), which also includes extracellular signal regulated kinase and p38-MAPK. As discussed above, autoantibodies to the proto-oncogene JUND in BC sera are also associated with the diagnosis of invasive BC. JUND, as well as c-JUN, are components of the AP-1 complex, a downstream target of JNK [94]. In support of the hypothesis that autoimmune tissue damage in BC leads to chronic inflammation and tumorigenic signals [9], JNK activation has been identified as a cellular response to environmental stresses, proinflammatory cytokines, and interleukins [95]. In addition, the JNK pathway may influence p53 and NF-κB pathways [96,97]. The JNK pathway has also been reported to have an important role in the control of cell survival and death pathways, and interference with the JNK pathway suppresses the induction of apoptosis by a variety of agents [98]. The results of Wang et al. demonstrated that GSTP1–1 has significant affinity for the C terminus of JNK and confirmed the ligand-binding regulatory role for this protein.

In summary, in addition to the recognized role of GSTP1 in detoxification and cancer susceptibility, autoantibodies to GSTP1 and to members of the AP-1 complex highly associated with the diagnosis of BC suggest that the immune system detects the participation of these signal transduction molecules in breast carcinogenesis.

#### Autoreactivity to the nDNA-encoded mitochondrial antigens PINK1 and DDOST confirms a link between Parkinson’s disease and breast cancer:

A rearrangement between the Pink1 AS ncRNA and a fragment of DDOST, a neighboring gene, was recognized by BC sera as an autoantigen (Table 1). The PTEN-induced kinase 1 (*PINK1*) gene encodes a serine/threonine protein kinase that localizes mainly to the mitochondrial inner membrane and is thought to protect cells from stress-induced mitochondrial dysfunction via the clearance of damaged mitochondria through selective mitophagy by mediating activation and translocation of PARKIN [99]. The *PINK1* gene is mutated in the germ line of some patients with hereditary early-onset Parkinson’s disease (PD) [100], and its pro-survival function on neuronal mitochondria has been related with the etiology of this disease. Widespread expression of natural antisense transcripts (NAT) has recently been suggested as a potential mechanism for providing regulatory complexity to the human ‘protein coding’ genome [101–103]. Scheele et al. reported that the human *PINK1* locus is regulated *in vivo* by a non-coding natural antisense RNA during modulation of mitochondrial function [104]. Although there are examples of NAT (coding or non-coding) that negatively regulate protein coding mRNA expression [105], few are associated with human disease [102]. Scheele et al. produced *in vivo* human data demonstrating that a non-coding RNA appears to ‘stabilize’ a sense coding RNA. Given the close genomic association between *DDOST* and *PINK1* in the human genome, they speculated that these two disease related genes may be uniquely co-regulated in humans. The NAT found in BC sera (Table 1) is compatible with this suggestion.

Berthier et al. reported that PINK1 protein is highly expressed in epithelial tissues such as in breast carcinomas and in the central nervous system. They concluded that PINK1 displays tissue-specific subcellular location and regulates apoptosis and cell growth in BC cells, indicating that the physiologic functions of PINK1 extends beyond its regulatory role of mitochondria-mediated cell survival in neurons [106].

O’Flanagan and O’Neill reviewed the function of PINK1 in cancer cell biology, with an emphasis on the mechanisms by which *PINK1* interacts with *PI3-kinase/Akt* signaling, mitochondrial homeostasis, and the potential context dependent pro- and anti-tumorigenic functions of *PINK1* [107]. Mechanistically, PINK1 interacts with the pivotal oncogenic PI3-kinase/Akt/mTOR signaling axis and controls critical mitochondrial and metabolic functions that regulate cancer survival, growth, stress resistance and the cell cycle [107]. The cytoprotective and chemo-resistant function for PINK1 has been highlighted by studies supporting *PINK1* as a target in cancer therapeutics. Previous work by Fernández Madrid et al. [16] identified ribosomal protein S6, an important component of *mTOR* signaling, as a BC autoantigen [16]. The study of Li et al. investigated the molecular mechanisms underlying the anti-BC effects of polyphyllin I, a natural compound extracted from *Paris polyphylla* rhizomes. They demonstrated that polyphyllin I induces mitophagy through DRP1-mediated mitochondrial fission and apoptosis. Notably, polyphyllin I treatment stabilizes PINK1 with its accumulation at the mitochondrial surface, which recruited PARK2 and led to mitophagy. *PINK1* knockdown combined with polyphyllin I treatment reduced mitophagy and enhanced DRP1-dependent fission, suggesting that PINK1 depletion leads to excessive fission and mitochondrial fragmentation [108].

On the other hand, little is known on the participation of *DDOST* in breast carcinogenesis. *DDOST*, dolichyl-diphosphooligosaccharide-protein glycosyltransferase non-catalytic subunit, is located in chromosome 1p36.12, a neighbor of *PINK1*; it encodes a component of the oligosaccharyl transferase complex that catalyzes the transfer of high-mannose oligosaccharides to asparagine residues on nascent polypeptides in the lumen of the rough ER. Enzymatic activity of this gene is involved in the processing of advanced glycation products, which develop from reactions between sugars and proteins or lipids and are associated with hyperglycemic phenotype and aging. Kim and Mak explored the hypothesis that cancer and PD may both result from *PTEN* dysregulation [109] and also suggested its involvement in both BC and Parkinson’s disease. Epidemiologic studies have suggested a link between PD and BC [110] but the nature of this association has remained elusive. The Danish population-based cohort study of Rugbjerg et al. identified 20,000 people with Parkinson’s disease over three decades from the National Danish Hospital Register. Patients were followed up for cancer in the Danish Cancer Registry, and their incidence rates of cancer were compared to age-, sex- and calendar period-specific rates in the general population as standardized incidence rate ratios. In a sub-analysis, they estimated the risk for cancer among patients with early onset PD and also compared breast tumor characteristics among women with PD to that of a control group. From data indicating that higher cumulative exposure of breast tissue to estrogens increases the risk for BC [111], and the suggestion that estrogens are neuroprotective and may protect against PD, as was proposed to explain the lower prevalence of Parkinson’s disease among women [112,113], the expectation was to find an inverse relation between PD and BC. However, Rugbjerg et al. found the opposite effect: the risk for female BC increased with increasing time since hospitalization for PD (Tables 3 and 5 in Rugbjerg et al. [110]). Thus, their results indicate a link with BC. They suggested that the observed increase in risk for BC of patients with PD might be due to factors other than estrogens and that the specific factors that cause the atypical cancer pattern of patients with Parkinson’s disease remain to be elucidated. In the work reviewed here the association with BC of autoreactive PINK1 and DDOST, its neighbor in the genome, confirms a link between PD and BC. This association is not an isolated finding since other autoreactive mitochondrial antigens found in BC sera are known to be involved in neurological diseases such as Alzheimer’s disease, autoimmune dementias, epilepsy, and other encephalopathies, suggesting that a panel of these autoantibodies can be biomarkers of both BC and neurological diseases.

In summary, *PINK1* plays an important regulatory role of mitochondria-mediated cell survival in neurons in association with PD and similarly it has a stabilizing and protective effect on mitochondria outside of the nervous system. It is likely that autoantibodies targeting PINK 1 and the rearrangement involving *PINK1* and its neighbor gene DDOST may be valuable biomarkers of BC risk as well as of PD.

#### SPATA5 participates in ribosome biogenesis and may be a biomarker for breast cancer risk:

The data on SPATA5 suggest that these autoantibodies may be biomarkers of BC risk but there is little evidence for any role in breast carcinogenesis. The protein encoded by the spermatogenesis associated gene *SPATA5* is a member of the AAA protein family defined by a highly conserved ATPase domain recognized by BC sera as an autoantigen (Table 1). Proteins belonging to this family function in multiple cellular processes that include membrane fusion, DNA replication, microtubule severing, protein degradation, and ribosome biogenesis [reviewed in 114]. The protein encoded by this gene has a putative mitochondrial targeting sequence and has been proposed to help maintain mitochondrial function and integrity during mouse spermatogenesis. Allelic variants in this gene have been associated with neurocognitive deficits.

Ribosome biogenesis is a very complex process indispensable for cell growth and division and is often upregulated in cancer cells, as it is a crucial determinant for fast growth, and therefore might provide a promising target for anti-tumor chemotherapy [114,115]. Initial reports linked SPATA5 to spermatogenesis [116] and depleting SPATA5 was found to affect profoundly mitochondria and neurons [117]. Other studies reported that SPATA5 is associated with the formation of the large 60S ribosomal subunit [118]. This is intriguing because the BC autoreactome identified several clones of autoantibodies to the 45S pre-ribosomal RNA associated with the diagnosis of invasive BC [unpublished], but presently there is no evidence that SPATA5 is associated with breast carcinogenesis.

#### Anti-mitochondrial antibodies targeting MFF and members of the JNK pathway suggest that the dynamic equilibrium between mitochondrial fusion and fission is disturbed in breast cancer:

The mitochondrial fission factor (*MFF)* gene encodes a protein that functions in mitochondrial and peroxisomal fission and recruits fission mediator dynamin-1-like protein (DNM1L) to the mitochondrial surface [119]. Mitochondrial fission is a complex process of vital importance for cell growth and survival. Mitochondria-shaping proteins such as MFF control the dynamic equilibrium between mitochondrial fusion and fission. The balance between fusion and fission is required to regulate mitochondrial quality control, cell metabolism, cell death, proliferation. and cell migration. There is also evidence that this dynamic equilibrium plays a role for the correct function of both the innate and the adaptive immune system [reviewed in 119]. Mitochondria undergo continuous fission and fusion to maintain their diverse cellular functions. Components of the fission machinery are partly shared between mitochondria and peroxisomes. Diseases associated with MFF include encephalopathy due to defective mitochondrial and peroxisomal fission and the co-occurrence of optic and/or peripheral neuropathy [120]. Among the pathways related to MFF are apoptosis and autophagy [121]. Several studies suggest that mitochondrial fission abnormalities are related to BC. IR-783 is a heptamethine cyanine dye that exhibits imaging, cancer targeting, and anticancer properties. There is evidence that its imaging and targeting properties are related to mitochondria [122]. Tang et al. reported that IR-783 induced the translocation of dynamin related protein 1 (DRP1) from the cytosol to the mitochondria, increased the expression of fission proteins, and decreased the expression of mitochondrial fusion proteins mitofusin1 and optic atrophy 1 (OPA1). They provided evidence suggesting that the anti-cancer properties of this compound are due to apoptosis induced by IR-783 in human BC cells by increasing mitochondrial fission. Studies in non-small cell lung cancer (NSCLC) have suggested a role of *JNK-MFF* signaling pathway in the carcinogenesis process [123]. LATS2, a tumor suppressor that affects NSCLC proliferation and mobilization, was found to be downregulated in A549 lung cancer cells. Overexpression of LATS2 induced apoptosis by activating mitochondrial fission via the *JNK-MFF* signaling pathway. Inhibition of the JNK pathway and/or knockdown of MFF abolished the pro-apoptotic effect of LATS2 on A549 cells [123]. The study of Seo et al. showed that protein isoforms of MFF, a molecule that controls mitochondrial size and shape, were overexpressed in patients with NSCLC and formed homo- and heterodimeric complexes with voltage-dependent anion channel-1 (VDAC1), a key regulator of mitochondrial outer membrane permeability [124]. The data reported identified the MFF–VDAC1 complex as a novel regulator of apoptosis with the potential to be exploited therapeutically.

The autoreactivity of MFF (Table 1) and that of JUND proto-oncogene AP-1 in BC sera suggest that the quality control mechanism responsible for mitochondrial fission is problematic in breast carcinogenesis.

## Conclusions

A group of phages cloned from BC cDNA expression libraries exhibited inserts identical to mitochondrial proteins encoded by nDNA, one of them being MNRR1. The evidence reported by Aras et al. suggested that MNRR1 regulates multiple genes that function in promoting the cell migration required for cancer metastasis and is upregulated in cell lines derived from tumors having enhanced metastatic potential. Since MNRR1 was identified as an autoantigen in breast carcinogenesis, and other mitochondrial autoantigens are potentially downstream of MNRR1, the data presented by Aras et al. support the role of MNRR1 and mitochondrial autoimmunity in breast carcinogenesis.

There is considerable evidence that the widespread autoreactivity of mitochondrial proteins found in BC sera may be the reflection of autoimmunity triggered by mitochondrial TAAs. Immune-screening of the RP cDNA library constructed with polyA RNA extracted from established BC cell lines and one pre-malignant cell, i.e., without the influence of immune cells or the society of cells found in the tumor microenvironment, led to the identification of a panel of nDNA-encoded mitochondrial proteins known to be involved in multiple tumorigenic pathways. The finding that these expression sequence tags identified in the RP cDNA library were significantly recognized by sera from patients with invasive BC indicates that the process that makes these proteins immunogenic takes place within the cancer cells themselves and constitutes direct evidence of the autoimmune nature of the reactivity.

Whether the function of enzymes targeted by autoantibodies is abnormal has been debated for decades [14,125]. The generation of autoreactive immune responses is not completely understood. Marré et al. hypothesized that pancreatic β-cell ER stress induced by environmental and physiological conditions generates abnormally modified proteins for the T1D autoimmune response [126]. Characterization of autoantigens has stimulated research on the pathogenesis of autoimmune diseases. For instance, most of the liver-specific autoantigens are key enzymes for the cell’s homeostasis. In patients with autoimmune hepatitis, autoantibodies can frequently be detected targeting cytochrome P450 2D6 (CYP2D6). Other members of the CYP family of enzymes have also been described as targets of liver-specific autoimmunity in several autoimmune liver diseases [127]. Again, how these enzymes become ‘self’ targets of autoantibodies is not yet established. There is much evidence that pathogenic antibodies are not the only agents responsible for autoimmune tissue damage. In primary biliary cholangitis, considered as a model autoimmune disease, the cloning of cDNAs encoding mitochondrial antigens led to the identification of the three enzymes of the 2-oxo-acid dehydrogenase family including pyruvate dehydrogenase (PDH). The autoantigen was found to be associated with the E2 subunit of these enzymes [11,128]. Further work on the pathogenesis of PBC showed that cell-mediated immune responses may be directly associated with the inflammatory response in PBC, suggesting that the generation of immune responsiveness to self-antigen can result in pathogenic autoimmune damage of tissues mediated by both humoral and cellular immune responses [129]. In this context, what is the evidence that the enzymes of the ETC targeted by autoantibodies may be dysfunctional? All the cloned sequences of the members of the ETC targeted by AMAs include conserved domains [17]. This finding and the high evolutionary conservation of the enzyme components of complex I may support the autoimmune nature of this process. In this light, Backes et al. provided evidence that evolutionary conserved proteins show specific sequence motifs and are more likely to become immunogenic [49].

The studies reviewed here demonstrate that the concept that MNRR1 directly participates in BC progression and metastasis can be extended to other autoreactive nDNA-encoded mitochondrial proteins that may contribute to mitochondrial dysfunction in BC, even if mitochondrial respiration is found to be within normal limits. Although a defect in oxidative phosphorylation may not be the cause of cancer as proposed by Warburg [75], the extensive autoreactivity of mitochondrial enzymes that has been demonstrated to be critical for the inception and progression of BC indicates that autoimmunity-induced dysfunctional mitochondrial proteins are critically involved in breast carcinogenesis and that at least Warburg was correct in implicating mitochondria in carcinogenesis.

The data suggesting that DDX21 orchestrates a complex signaling network, with participation of JUND and ATF3 driving chronic inflammation and breast tumorigenesis, illustrate that functionally the division of mitochondrial proteins into mtDNA- and nDNA-encoded is somewhat arbitrary in this case, since DDX21 is a nucleolar protein that can translocate to the nuclei as well as to the mitochondria, whereas although JUND and ATF3 are strongly expressed in the mitochondria they are predominantly located in the nuclei.

That key mitochondrial enzymes involved in energy metabolism such as GADPH and PKM2 are targeted by autoantibodies in BC makes the important point that, even if other parameters fail to show mitochondrial dysfunction, these two mitochondrial proteins are clearly involved in breast carcinogenesis. In addition, besides MNRR1, a number of other nDNA-encoded mitochondrial proteins such as GSTP1, PINK1, SPATA5 and MFF have been implicated in BC progression or BC risk, suggesting that mitochondrial autoimmunity prominently participates in breast carcinogenesis.

The evidence of participation of mitochondrial autoimmunity in BC inception and progression reviewed here may challenge the prevailing thinking in oncogenesis and suggests that mitochondrial autoimmunity is a major contributor to breast carcinogenesis and probably to the inception and progression of other solid tumors.

**Figure 4:**
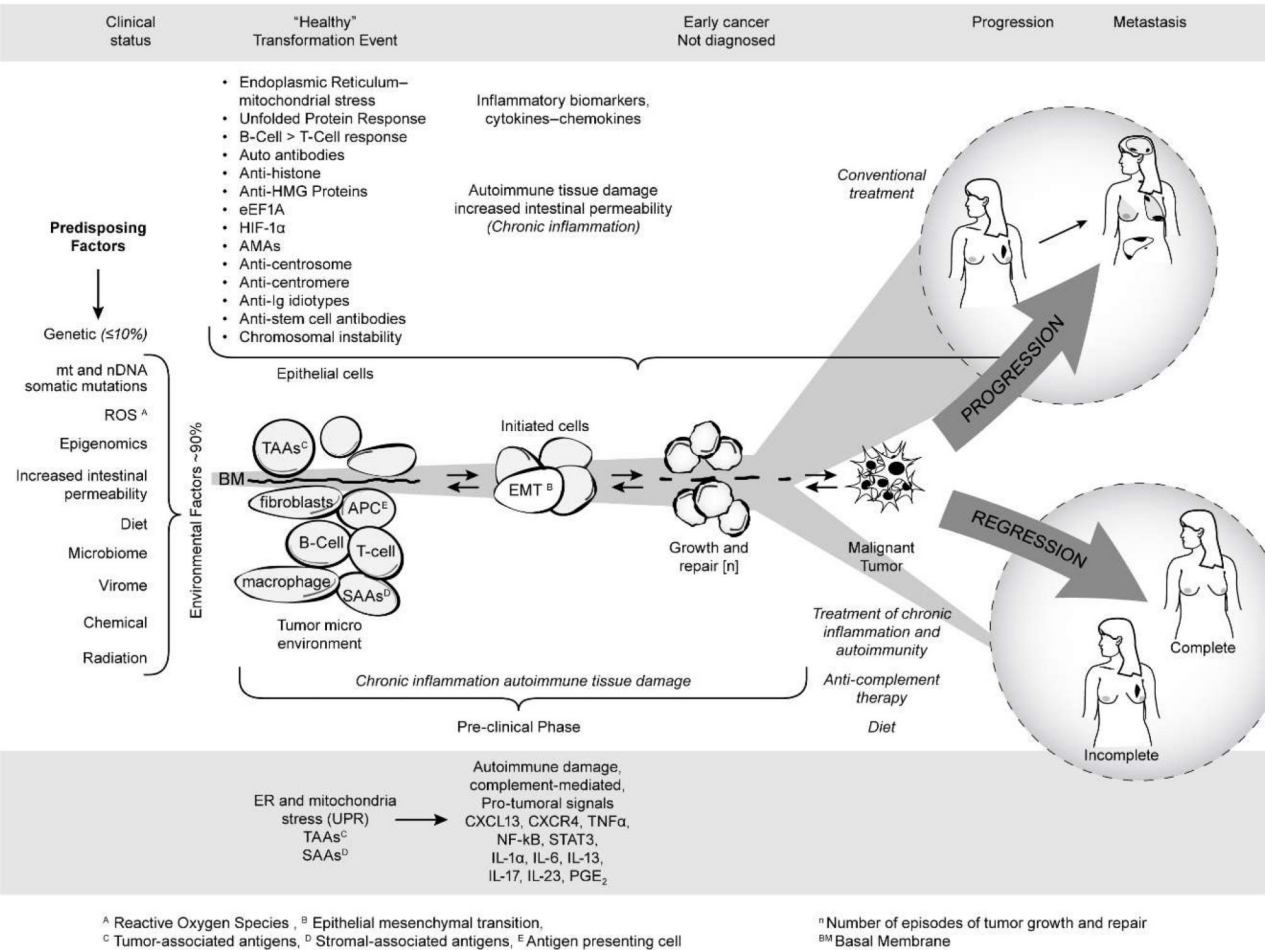
Breast cancer as an autoimmune disease.

Figure 4 summarizes the published and unpublished work reviewed here. It shows that mitochondrial autoimmunity is a major factor in cancer inception and progression but by no means the only factor since there is a multiplex autoantibody profile targeting centrosome and stem cell antigens as well as anti-idiotypic antibodies revealing the complex signaling network involved in breast carcinogenesis. The autoreactome also sheds light on predisposing factors for sporadic BC including ROS, epigenomics, increased intestinal permeability, the microbiome, and diet. The barrage of autoantibodies and inflammatory cytokines can create a chronic inflammatory milieu with generation of tumorigenic signals leading to BC. The diagram also shows that the course of cancer could potentially be prevented if detected early. The studies reviewed here open new, unexpected therapeutic avenues and possibilities for cancer prevention and treatment of patients with cancer. These avenues are derived from an entirely new perspective of breast carcinogenesis based on existing therapeutic tools that have been successfully used in the treatment of the autoimmune RADs as well as on future measures that will be developed to prevent or avoid the effect of pathogenic antibodies and inflammatory cytokines on target organs. It is likely that the addition of modalities such as protecting the integrity of mitochondria, the induction of tolerance to antigens critically targeted by the autoantibody response, selective suppression of B-cell hyperactivity, anti-inflammatory therapy including anti-inflammatory cytokine treatment, and drugs that inhibit complement-induced autoimmune damage, combined with appropriate diet, may improve the prognosis and decrease the mortality of aggressive BC.

The body of work reviewed here also questions the criteria used to classify a disease as autoimmune as applied to cancer [130,131]. Autoimmune diseases in which pathogenic antibodies have been identified can be organ-specific when the autoimmune process targets predominantly or exclusively one organ as in type 1 diabetes mellitus, autoimmune thyroid diseases such as Hashimoto’s thyroiditis, and Graves’ disease, or one tissue in particular such as the neuromuscular junction in the nervous system. But autoimmune diseases can also be systemic as in SLE, RA, or Sjögren’s syndrome, in which there are many pathogenic antibodies in addition to the participation of inflammatory cytokines and cellular mechanisms. Several features in the autoantibody profile are similar in cancer and in the autoimmune diseases, suggesting that some common pathogenic mechanisms can lead to their development. Similarly, there is much evidence for some commonalities in the molecular mechanisms in different solid tumors. In BC the multiplicity of autoantibodies highly associated with this malignancy approaches the large numbers of autoreactive gene products identified in the systemic autoimmune diseases such as SLE [14,132]. For example, it is clear that the breast is the main target of autoimmunity leading to autoimmune tissue damage in the breast and to cancer development; in that sense, BC could be regarded as a predominantly organ-specific autoimmune disease. Systemic aspects of the disease are suggested by the nature of the targets identified by autoantibodies including antigens known to participate in maintaining the integrity of the intestinal epithelial barrier, in the pathogenesis of some neurologic diseases, and antigens reflecting the interaction of the immune system with bacterial and viral products of the microbiome [unpublished]. In addition to pathogenic antibodies and inflammatory cytokines, similarly to the RADs, other mechanism may potentially be responsible for autoimmune tissue damage dependent on immune cell infiltration and B cell expansion in the target tissue [61,133–135].

## Figures and Tables

**Table 1:** Panel of nDNA-encoded mitochondrial proteins recognized as autoantigens that may be involved in breast carcinogenesis.

Antigen	E value/%ID	(p) BC/Libr.	Accession/ Chromosome
DDX21	2E-96/100	1.60E-16/RP	NM_004728.4/10q22.1
GAPDH	5e-81/100	0.001/RP	NR_152150.2/12p13.31
PKM2	9E-149/100	0.001/RP	NM_001206779.2/15q23
GSTP1–1	3E-70/100	0.02/RP	NM_000852.4/11q3.2
SPATA5	4E-102/100	4.94E-06/RP	XM_017007827.2/4q26.1
MFF	4E-38/100	0.001/B	NM_00127706.2/2q36.3
*ncRNA PINK1-AS/DDOST*	9E-126/100	2.61E-05/RP	NR_046507.1/1q36.12

DDX21: DEAD-Box helicase 21; GAPDH: Glyceraldehyde 3-phosphate dehydrogenase; PKM2: Pyruvate Kinase isoenzyme M2; GSTP-1: Glutathione S-Transferase Pi 1; SPATA5: Spermatogenesis Associated 5; MFF: Mitochondrial Fission Factor; ncRNA: non-coding RNA; PINK1-AS/DDOST: PTEN Induced Kinase 1 Anti-sense/Dolichyl-diphos-phooligosaccaride protein
